# Systematic Error in Gaming Disorder Measures

**DOI:** 10.1192/bjp.2024.241

**Published:** 2025-10-30

**Authors:** Veli-Matti Karhulahti, Tiina Auranen

**Affiliations:** 1 University of Jyväskylä, Seminaarinkatu 15, PL 35, 40014, Finland

The inclusion of gaming disorder to the ICD-11 as a new addictive behavior was supported by epidemiological estimates, which have implied such psychiatric problems to be globally prevalent (1). After nosological criteria for diagnosing gaming disorder were proposed in the DSM-5 and the ICD-11, studies have collected further epidemiological evidence by using more diagnostically consistent self-report measures across countries and populations (2). According to recent meta-analyses that include mixed measures, the global prevalence of gaming disorder is 2–3 % (3). This letter reports a sequence of events that can significantly affect the interpretation of such epidemiological rates.

As part of the participant recruitment of our longitudinal research, we surveyed the general Finnish population to identify people who have sought clinical treatment for gaming disorder symptoms. Gaming disorder criteria from the ICD-11 were used for prescreening. We reached 44 people who self-reported treatment-seeking due to gaming, seven of which contacted us and expressed interest to join the study. In the entrance interview with a clinician, however, it turned out that six out of the seven people had not sought treatment for gaming but *gambling*.

This led us to closely reinvestigate the content of validated gaming disorder measures, which are based on the DSM-5, the ICD-11, and other nosological systems. Surprisingly, we did not find any of the validated English DSM-5 and ICD-11 based gaming disorder self-report measures (2) nor those listed as the most used measures (3) having items or instructions that would exclude gambling. Because these measures ask about behaviors in digital games (i.e., computer games, internet games, video games), and many gambling products are digital games too, people with *gambling disorder* symptoms can also meet the criteria of *gaming disorder* measures.

The above has not been a problem in gambling measurement because gaming conceptually includes gambling but not *vice versa*: in English, all gambling is gaming but not all gaming is gambling (Fig 1). In other languages the clinical associations between gambling and gaming terminologies vary (4) and would benefit from systematic global assessment.

Our findings imply that most, if not all, epidemiological estimates of gaming disorder combine both gaming *and* gambling prevalence rates. Considering that the global prevalence of clinical gambling problems has been estimated to be 0.12−5.8 % (5), the present finding should encourage researchers to critically reconsider the current epidemiological knowledge produced by gaming disorder self-report measures and meta-analyses. This can also help explain previously found correlations between gaming and gambling related health problems (6).

Next, researchers should carefully investigate the prevalence of gambling exclusion efforts in gaming disorder measurement across languages, populations, and specific scales to produce more reliable epidemiological estimates. This will be helpful to further improve both policy and practice globally as well as regionally. The developers of new gaming disorder screening measures should, by default, involve instructions or items that explicitly tell participants to not report their gambling behaviors.

## Figures and Tables

**Figure 1:**
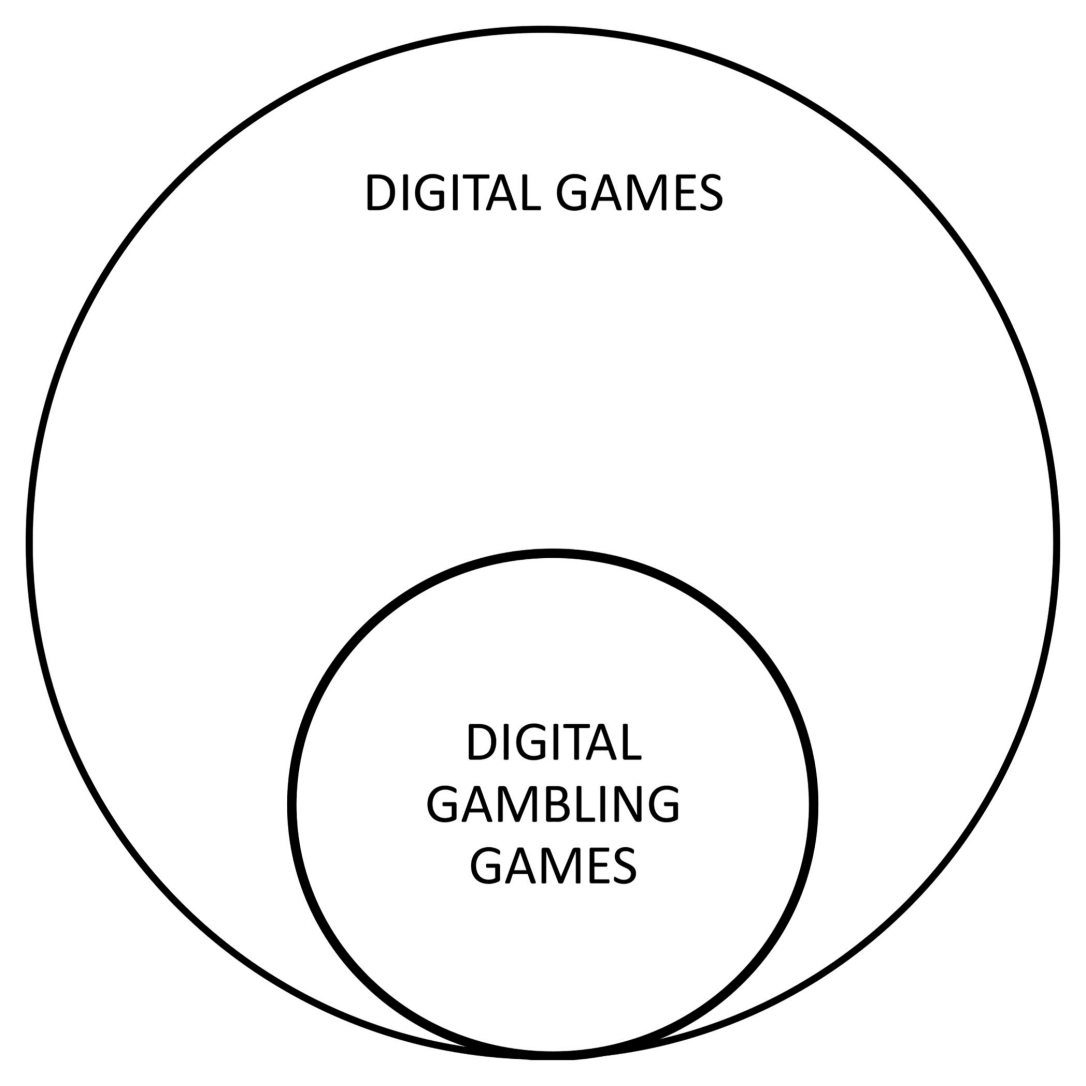
Conceptual overlap in English.
